# Choline Intake as Supplement or as a Component of Eggs Increases Plasma Choline and Reduces Interleukin-6 without Modifying Plasma Cholesterol in Participants with Metabolic Syndrome

**DOI:** 10.3390/nu12103120

**Published:** 2020-10-13

**Authors:** Marissa DiBella, Minu S. Thomas, Hana Alyousef, Courtney Millar, Christopher Blesso, Olga Malysheva, Marie A. Caudill, Maria Luz Fernandez

**Affiliations:** 1 Department of Nutritional Sciences, University of Connecticut, Storrs, CT 06269, USA; Marissa.dibella@uconn.edu (M.D.); minu.thomas@uconn.edu (M.S.T.); hana.alyousef@uconn.edu (H.A.); Courtneymillar@hsl.harvard.edu (C.M.); Christopher.blesso@uconn.edu (C.B.); 2 Division of Nutritional Sciences, Cornell University, Ithaca, NY 14853, USA; ovm4@cornell.edu (O.M.); mac379@cornell.edu (M.A.C.)

**Keywords:** eggs, choline supplement, plasma cholesterol, plasma choline, Vitamin E, selenium

## Abstract

Metabolic syndrome (MetS) is characterized by low-grade inflammation and insulin resistance, which increase the risk of heart disease. Eggs have numerous nutrients including choline, carotenoids, and fat-soluble vitamins that may protect against these conditions. Egg phosphatidylcholine (PC) is a major contributor of dietary choline in the American diet. However, uncertainty remains regarding eggs due to their high concentration of cholesterol. In this study, we evaluated the effect of two sources of choline, whole eggs (a source of PC) and a choline supplement (choline bitartrate, CB), on plasma lipids, glucose, insulin resistance, and inflammatory biomarkers. We recruited 23 subjects with MetS to participate in this randomized cross-over intervention. After a 2-week washout, with no choline intake, participants were randomly allocated to consume three eggs/day or CB (~400 mg choline/d for both) for 4 weeks. After a 3-week washout period, they were allocated to the alternate treatment. Dietary records indicated higher concentrations of vitamin E and selenium during the egg period (*p* < 0.01). Interestingly, there were no changes in plasma total, low density lipoprotein (LDL)- or high density lipoprotein (HDL)-cholesterol, triglycerides, or glucose, compared either to baseline or between treatments. In contrast, interleukin-6 was reduced, with both sources of choline compared to baseline, while eggs also had an effect on lowering C-reactive protein, insulin, and insulin resistance compared to baseline. This study demonstrates that in a MetS population, intake of three eggs per day does not increase plasma LDL cholesterol, and has additional benefits on biomarkers of disease compared to a choline supplement, possibly due to the presence of other antioxidants in eggs.

## 1. Introduction

Metabolic syndrome (MetS) is characterized by dyslipidemia, hypertension, insulin resistance, and large waist circumference, which further increases risk for development of cardiovascular disease (CVD) and type 2 diabetes [1]. Eggs are a nutrient dense, low-cost food that provides key nutrients such as choline, vitamin D, riboflavin, vitamin B12, lutein, and zeaxanthin to the diet [2]. Eggs contain a favorable ratio of unsaturated to saturated fatty acids (2:1 per 100 g whole egg). One large egg contains only 70 calories and provides 6 g of protein, which can lead to prolonged satiety [3,4]. Since carbohydrate restricted diets have been associated with improved markers of MetS, the low carbohydrate content in eggs could be beneficial to this population [5].

The question of whether dietary cholesterol negatively impacts plasma cholesterol concentrations and lipoprotein profiles continues to be a heated debate within the research community. Eggs have been controversial for a long time due to their high cholesterol content. While some research reports that dietary cholesterol or egg consumption may increase the risk of CVD [6,7], the majority of current research does not support this claim. Several clinical trials have demonstrated that egg consumption favorably increases plasma high density lipoprotein cholesterol (HDL-C), and improves plasma ratios of low density lipoprotein cholesterol (LDL)-C/HDL-C [8,9]. Additionally, studies report that dietary cholesterol in general does not negatively impact plasma cholesterol concentrations or LDL-C/HDL-C ratio [4,8,9,10,11,12]. According to the American heart association, eggs are not significantly associated with risk of CVD, although some meta-analysis have reported that in some cases excess dietary cholesterol has been associated with elevations in LDL-C [13]. The low saturated fat content and high concentrations of other beneficial nutrients in eggs may account for the lack of association between eggs and heart disease, despite their high cholesterol content [11,14]

Cholesterol homeostatic mechanisms have been known for many years [15]. Previous studies have demonstrated that these mechanisms allow for the control of plasma cholesterol levels in most, but not all, subjects, when dietary cholesterol intake is increased [16]. One such mechanism is the reduction in the transcription of cholesterol synthesizing enzyme 3-hydroxy-3-methylglutaryl CoA reductase (HMG-CoA reductase) [11,15]. The downregulation of HMG-CoA reductase leads to the decrease in endogenous cholesterol synthesis, to counteract the increase in exogenous cholesterol intake [17]. An upregulation of bile acid synthesis has also been reported in response to higher dietary cholesterol intake. The formation of bile acids, and their excretion through the feces, result in a decrease in plasma cholesterol concentration, counteracting the increased dietary intake [18]. Therefore, the body can adapt to the dietary intake of cholesterol, preventing a fluctuation in plasma cholesterol levels with increased dietary cholesterol intake.

A recent study found no change in plasma LDL-C concentration in a MetS population consuming three whole eggs/day vs. a yolk-free substitute for 12 weeks [8]. Conversely, an increased prevalence of large LDL particles and a decrease in small LDL particles was also reported [8]. Therefore, egg yolk, although high in cholesterol, is considered to have beneficial health effects on lipoprotein profiles in people with MetS, potentially due to both, the formation of less atherogenic LDL particles [9], and their high phosphatidylcholine (PC) concentration [19]. Recently, the Dietary Guidelines for Americans (2015–2020) removed the recommendation to limit dietary cholesterol to ≤300 mg/day [20]. Regardless, due to their high cholesterol content, people with MetS continue to be cautious regarding egg intake.

Choline is an essential nutrient that is critical for cell structure and signaling [21]. Choline from eggs has been shown to protect against inflammation in human and animal studies [19]. Furthermore, dietary choline was inversely associated with inflammatory markers in the ATTICA study [22]. Research shows that most United States adults do not achieve adequate intake (AI) of choline [23]. A large egg provides 164 mg of choline, which corresponds to 30% and 40% of the recommended intakes for men (550 mg/day) and women (425 mg/day), respectively [24].

With controversy continuing to surround the inclusion of eggs in a healthy diet, the aim of this study was to further evaluate the effect of egg consumption on cholesterol concentrations, choline concentrations, and insulin resistance in a MetS population. A previous study in a healthy, young population reported that the consumption of three eggs/day for 4 weeks resulted in higher plasma choline concentration, when compared with intake of choline bitartrate supplements [25]. Based on this study, we hypothesized that, compared to choline bitartrate supplement, consumption of PC, as present in eggs, would result in higher plasma choline concentrations, and therefore would have a more favorable effect on inflammatory markers in people with MetS.

## 2. Materials and Methods

### 2.1. Experimental Design

Twenty-three men and women aged 35–70 years with MetS were recruited to participate in this randomized cross-over study. The primary outcome variable of this study was plasma choline. Based on the standard deviation from previous studies, where increases in choline were observed following egg consumption [8,25], and using a Z value of 1.96 (95% confidence interval), we estimated that 20 subjects would be sufficient to observe differences between groups. Thus, we recruited 23 subjects to allow for attrition. This study was powered to detect a 7% change in plasma choline concentration at 80% power, with *p* < 0.05.

MetS was defined according to the National Cholesterol Education Program guidelines that consists in having 3 of the following biomarkers: blood pressure (BP) ≥130/85 mmHg; fasting glucose ≥100 mg/dL; triglycerides (TG) ≥150 mg/dL; waist circumferences: males ≥102 cm, females ≥88 cm; HDL cholesterol: males ≤40 mg/dL, females ≤50 mg/dL. Exclusion criteria were BP >140/100 mmHg; triglycerides (TG) >500 mg/dL; fasting glucose >126 mg/dL; total cholesterol >240 mg/dL. Other exclusion criteria included having liver or renal disease, diabetes, cancer, heart disease or a history of stroke, being vegan or vegetarian, taking choline supplements, or having allergies to eggs.

Once qualified, participants began a 2-week washout period where they abstained from eating eggs. Following a baseline visit, participants were randomly allocated to a 4-week intervention of either 3 eggs per day (Egg) or choline supplements (choline bitartrate, CB). Both treatments consisted in ~400 mg choline/day. Following a 3-week washout period, subjects were allocated to the alternate treatment. Large eggs were purchased from the local grocery store, and egg supply was provided for two weeks at a time. CB supplement was given in full at the beginning of the supplement intervention. The supplement was obtained from Best Naturals (Kenilworth, NJ, USA), and participants were instructed to take 1.5 tablets/day. Participants were told to consume eggs or supplements during the morning for breakfast. No guidelines were given on preparation of eggs. Compliance was self-reported each week.

All participants signed an informed consent before joining the study. The study protocol was approved by the Institutional review board of the University of Connecticut. This clinical trial was registered to Clinical.trials.gov (Protocol NCT03877003).

### 2.2. Dietary and Exercise Records

Participants were asked to complete 3-day diet and exercise records during the washout periods and during each intervention period for a total of four sets. Records were self-reported and reviewed with the researcher at each data collection point to allow for clarifying questions. Diet records were analyzed using Nutrition Data Systems for Research software (NDSR) (Minneapolis, MN, USA). Nutrient reports were averaged for each phase. Exercise records were self-reported on concurrent days. Records were analyzed for consistency between study phases.

### 2.3. Anthropometric Measurements and Blood Pressure

Height, weight, and waist circumference were measured at screening and at all 4 consecutive visits including baseline, after the washout period (second baseline), and at the end of each intervention. Height (cm) was measured on a wall mounted stadiometer, and weight (kg) on a digital scale. Waist circumference was measured with a flexible tape measure at the top of the iliac crest, or over the navel point if the iliac crest could not be located. An average of three readings were recorded. Blood pressure was the average of three consecutive readings, measured using an automatic blood pressure machine (Omron, Bolingbrook, IL, USA).

### 2.4. Plasma Lipids, Glucose, C-Reactive Protein (CRP), Creatinine, and Liver Enzymes

Antecubital venous blood samples were collected in EDTA-coated vacutainer tubes. Samples were centrifuged at 2000× *g*, and plasma was collected for analysis. Plasma HDL cholesterol, total cholesterol, triglycerides, glucose, CRP, and liver enzymes were measured using a Cobas C 111 analyzer (Roche Diagnostics, Indianapolis, IN, USA). LDL cholesterol was calculated using the Friedewald equation as previously reported [8]. All these analyses were conducted at 4 points of time, as described above.

### 2.5. Plasma Choline

Plasma choline was measured in duplicate by liquid chromatography coupled with tandem mass spectrometry (LC-MS/MS), as previously described [26]. Briefly, plasma samples were mixed with acetonitrile containing 0.1% formic acid and d13 choline as an internal standard; the supernatant was collected, mixed with acetonitrile containing 0.1% formic acid, and injected into the LC–MS/MS system. The inter-assay Coefficient of Variance was <3.5% for each metabolite, based on duplicate measures and <5% based on in-house controls. Analysis for plasma choline was done at 4 points as described above.

### 2.6. Plasma Insulin and Insulin Resistance

Plasma insulin was assessed using a sandwich enzyme-linked immunosorbent assay (ELISA), which uses antibodies to capture insulin and chromogenic substrate for detection by a plate reader. Each sample was analyzed in duplicate. Insulin resistance was calculated using the HOMA-IR formula: fasting insulin (pg/mL) × fasting glucose (mg/dL)/22.5 [27]. Due to miscalculations in blood collection after the washout period, we did not have enough plasma to measure insulin after the washout, therefore we only have 3 points for insulin; the beginning of the study and at the end of each intervention.

### 2.7. Inflammatory Markers

A Magnetic Luminex Performance Assay multiplex kit (R&D Systems Inc., Minneapolis, MN, USA) was used to measure interleukin (IL)-6, tumor necrosis factor (TNF)-α, and monocyte chemoattractant protein (MCP)-1 simultaneously. The assay uses a magnetic, antibody-coated plate that contains fluorophores into which samples and microparticles are pipetted. Immobilized antibodies and fluorophores in plate wells bind the analyte of interest so that, when analyzed by the Luminex system, the presence and magnitude of each cytokine is concurrently measured, as previously reported [28]. These analyses were also conducted at the beginning of the study and at the end of each intervention, for a total of 3 points for the inflammatory markers, due to insufficient plasma collected after the washout.

### 2.8. Statistical Analysis

A paired *t*-test was used to evaluate differences in dietary records between the egg and the supplement period. Repeated measures ANOVA was used to analyze plasma lipids, glucose, CRP, liver enzymes, inflammatory markers, insulin, insulin resistance, and choline, with time being the repeated measure, and group being the eggs vs. the supplement. *p* < 0.05 was considered to be significant.

## 3. Results

### 3.1. Dietary and Exercise Records

Diet and exercise records indicated that total energy intake (Kcal/day) and exercise (min/day) were consistent throughout the 13 weeks. There were no statistical differences in macronutrient or micronutrient intake between baselines (Data not shown). Fat and protein intake were greater during the egg, compared to CB supplement, phase. Conversely, carbohydrate intake was lower during the egg phase compared to the CB supplement phase (*p* < 0.001) (Table 1).

Cholesterol and monounsaturated fatty acid (MUFA) intakes were also greater during the egg phase, as where Vitamin D and selenium intake (*p* < 0.001); in agreement with the nutrients present in egg. Fiber intake was consistent across intervention periods (Table 1).

### 3.2. Initial Characteristics of Subjects

Initial characteristics of subjects with metabolic syndrome are presented in Table 2. Although the mean values for systolic blood pressure and plasma triglyceride are lower than the definition for metabolic syndrome, individual values were >150 mg/dL or >135 mm Hg (data not shown).

### 3.3. Plasma Choline Concentrations

**Figure 1:**
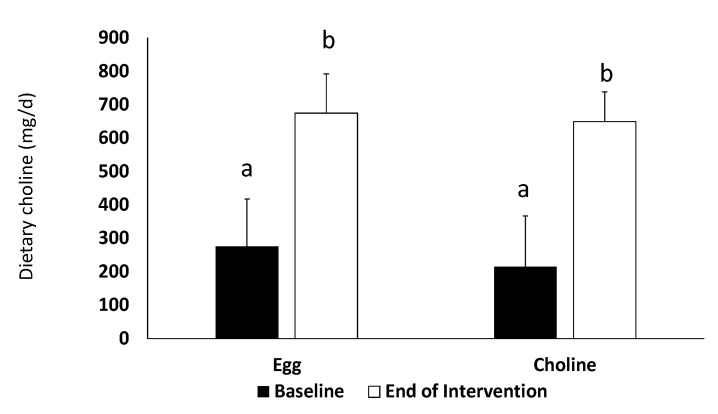
Dietary intake of choline at baseline (dark bar) and at the end of each intervention (white bar) for subjects after the egg and the CB treatment. Different superscripts (a, b) indicate significantly different from each other (*p* < 0.001). Data are presented as mean + SD for *n* = 23 subjects.

As expected, choline intake significantly increased from baseline to end of intervention for both egg and supplement periods (*p* < 0.001) (Figure 1).

### 3.4. Anthropometrics, CRP, and Liver Enzymes

Body weight, body mass index, and waist circumference remained constant throughout the study indicating no fluctuations in weight during the intervention. There was also no change in systolic or diastolic blood pressure or liver enzymes throughout the study (Table 3). However, there was a decrease in CRP, from baseline to end of the egg period (*p* < 0.05), which was not observed after supplement intake (Table 3).

### 3.5. Plasma Lipids and Glucose

There were no significant changes in plasma total cholesterol, LDL cholesterol, HDL cholesterol, or LDL/HDL ratio throughout the study (Table 4). There was also no change in plasma triglycerides or fasting plasma glucose.

### 3.6. Plasma Choline

**Figure 2:**
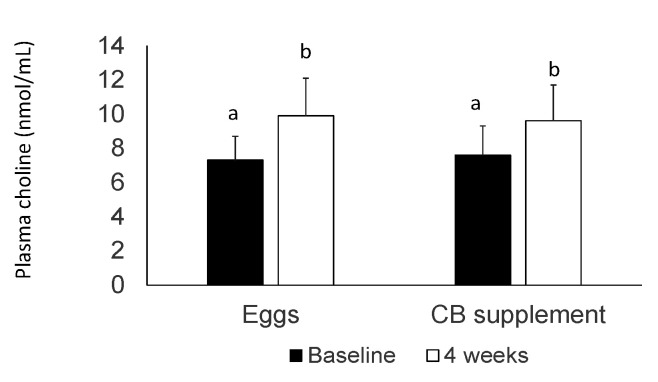
Concentrations of plasma choline at baseline (dark bar) and following the intervention (white bar). Different superscripts (a, b) indicate significantly different from each other (*p* < 0.001). Data are presented as mean + SD for *n* = 23 subjects.

In agreement with choline intake, fasting plasma choline increased from baseline to end of intervention after both interventions (*p* < 0.01), with no significant changes between treatments (Figure 2).

### 3.7. Inflammatory Markers

Plasma concentrations of IL-6 decreased from baseline to the end of the intervention for both dietary treatments (*p* < 0.01). There was no change in plasma concentrations of TNF- α or MCP-1 throughout the study. Plasma insulin concentrations were lower after the egg phase compared to baseline. There were no differences in plasma insulin at the end of both interventions (Table 5). Similarly, insulin resistance measured by HOMA-IR only decreased in eggs compared to baseline, but there was no significant difference between eggs and supplement at the end of each intervention.

## 4. Discussion

In this study, we demonstrated that, contrary to what we hypothesized based on previous research [25], there were no differences in plasma choline concentrations when we compared intake of egg versus supplement in this group of subjects with MetS. Both groups experienced increases in plasma choline, independent of the source of choline. These increases in choline may be responsible for the observed decreases in IL-6, since choline has demonstrated anti-inflammatory effects [19]. A very interesting finding was that there were no changes in total cholesterol, HDL-C, LDL-C, HDL/LDL ratio, triglycerides, or fasting blood glucose even after the consumption of an additional 550 mg of dietary cholesterol coming from eggs. Furthermore, we observed that insulin, insulin resistance, and C-reactive protein (CRP) were lower during egg intake compared to baseline, an effect not seen with the choline supplement.

Previous studies found dietary choline intake to be negatively associated with insulin resistance (HOMA-IR) [29]. In the present study, the participant’s dietary choline intake was significantly increased compared to baseline when choline intake was below AI for all subjects. During intervention phases, participants were on average consuming above the AI levels for choline. Findings from the present study suggest that adequate choline intake from eggs may improve insulin concentrations and HOMA-IR in people with MetS. Plasma choline concentrations increased from baseline to end of both interventions, but there was no difference when comparing end of egg phase to end of supplement phase. This is a surprising finding because our previous research found that three eggs/day for 4 weeks increased plasma choline when compared to CB in a healthy, young population [25]. This raises questions about how choline is absorbed in people with MetS. It is well established that lysophosphatidylcholine and free choline are absorbed differently [19]. It is possible that MetS may influence free choline-mediated absorption through the CT-L1 transporter, which has been shown to be responsive to corticosteroids [30]. MetS is characterized by elevated glucocorticoid activity [31], and therefore increased absorption through CTL-1. This increased absorption may have led to similar absorption rates for both PC (as is present in eggs) and free choline (as present in the supplement), resulting in similar increases in plasma choline after both interventions. Further research is needed to understand the mechanisms of choline absorption in people with metabolic syndrome.

The key changes in macronutrient composition during the egg period, mainly lower carbohydrate, higher fat and protein, and higher MUFA were due to the inclusion of three eggs/day during breakfast. Furthermore, during the egg intervention phase, participants also had higher intake of dietary vitamin D and selenium compared to the supplement phase. Low vitamin D status has been associated with insulin resistance in men and lower HDL cholesterol levels in women [32]. Selenium supplementation has been shown to improve insulin concentrations and HOMA-IR in people with type 2 diabetes and coronary heart disease [33]. The dietary intake of these two nutrients from eggs may explain the improvement of HOMA-IR and insulin concentrations observed during the egg phase compared to baseline.

Dietary cholesterol intake increased during the egg phase, but total plasma cholesterol, HDL, LDL, and HDL/LDL cholesterol ratio did not change. Similar findings of no change in total cholesterol or LDL cholesterol were previously observed in subjects with MetS [28]; however, there was a significant increase in HDL cholesterol in these subjects. It is possible that the lack of changes observed in this study might be associated with the fact that this was not a weight loss intervention, as it is well-established that weight loss results in significant improvements in lipoprotein profiles [34,35].

Several studies have reported no effect of egg consumption on plasma triglycerides, a biomarker of MetS. For example, intake of three eggs/day for 4 weeks did not increase plasma triglycerides [9]. In agreement with these findings, our study showed that consuming three eggs/day for 4 weeks did not change plasma triglyceride levels in people with MetS. This finding expands on previous claims about egg consumption, and further demonstrates that eggs do not negatively impact triglyceride levels.

Previous findings about the effects of egg consumption on fasting blood glucose levels have been mixed. While some studies have reported higher egg consumption to be associated with higher blood glucose in people with impaired glucose tolerance [36], other research shows that egg consumption can improve blood glucose levels in people with pre-diabetes and type II diabetes [37,38]. The current study found that egg consumption did not negatively affect fasting blood glucose in people with MetS. During the egg phase, participants consumed fewer carbohydrates (% energy) and more protein (% energy) compared with the supplement phase. This change in macronutrient composition of the diet, which occurred while participants were eating three eggs/day, may help to explain how egg intake was beneficial to blood glucose concentrations.

Metabolic syndrome can often be characterized by chronic low-grade inflammation [39]. In this study, inflammation was assessed by serum levels of MCP-1, TNF-α, IL-6, and CRP. These inflammatory biomarkers were analyzed at baseline and after each intervention to understand the effect of egg consumption on inflammation status in people with MetS. While no significant change in plasma TNF-α or MCP-1 was observed, there was a significant decrease in IL-6 from baseline after both interventions. Previous research has shown higher choline intake to be associated with lower IL-6 concentrations in healthy adults [22]. The current study’s findings may indicate a similar effect to be true in people with MetS. CRP is a well-accepted biomarker of inflammation that is often elevated in people with MetS, and is a predictor for risk of CVD [40]. Previous research has demonstrated that intake of three eggs/day for 12 weeks reduces CRP levels in healthy, overweight individuals. This finding was attributed to an increase in serum adiponectin and a reduction in adipose tissue due to weight loss [41]. In our study, a decrease in CRP was observed only after the consumption of three eggs/day for 4 weeks. This occurred even in the absence of weight loss and can be attributed to other antioxidants present in eggs.

There is one limitation to this study that needs to be discussed, mainly the small number of individuals who completed the intervention, which could have a bearing on the results of plasma lipids. The lack of a significant difference in plasma cholesterol, LDL, and HDL-C was surprising since we have previously demonstrated in other populations, increases in these parameters following egg consumption [42,43,44]. Our previous study, with metabolic syndrome patients during a weight loss intervention, also showed no increases in LDL-C after consuming three eggs, however HDL-C did increase significantly [8]. Additional studies with metabolic syndrome patients during weight maintenance are needed to further clarify how eggs modify lipoprotein metabolism in this population.

## 5. Conclusions

In conclusion, three eggs/day did not increase biomarkers for CVD, specifically LDL cholesterol, in people with MetS. Moreover, eggs did not negatively affect any of the biomarkers of MetS, while improving the inflammatory marker IL-6. Eggs also improved HOMA-IR and decreased plasma insulin. Eggs are a good source of choline thus consumption of eggs enables individuals to achieve adequate intake of this nutrient. Therefore, whole eggs could be considered a healthful food choice for people with MetS.

## Figures and Tables

**Table 1:** Comparison of macronutrients, cholesterol, choline, dietary fiber, and carotenoids between the egg and choline bitartrate (CB) supplement periods ^1^.

Parameter	Egg	CB Supplement
Energy (Kcal/day) ^2^	1860 ± 152	1675 ± 141
Fat (% Energy)	**42.4 ± 7.6 ^a^**	**36.4 ± 6.1 ^b^**
Carbohydrate (% Energy)	**34.9 ± 10.0 ^a^**	**44.1 ± 9.2 ^b^**
Protein (% Energy)	**20.3 ± 5.2 ^a^**	**17.6 ± 6.0 ^b^**
Alcohol (% Energy)	2.3 ± 4.2	1.9 ± 3.2
SFA (g/d)	30.8 ± 14.3	26.0 ± 13.6
MUFA (g/d)	**31.2 ± 11.2 ^a^**	**24.3 ± 10.9 ^b^**
PUFA (g/d)	17.2 ± 8.8	15.2 ± 7.5
TFA (g/d)	2.1 ± 1.1	2.0 ± 1.0
Omega (g/d)	2.0 ± 1.0	1.9 ± 1.1
Added sugar (g/d)	31.9 ± 32.8	41.1 ± 32.0
Glycemic index	59.0 ± 6.9	57.8 ± 5.4
Glycemic load	94.8 ± 55.8	100.7 ± 49.5
Fiber (g/d)	16.0 ± 6.8	17.6 ± 5.2
Soluble Fiber (g/d)	5.8 ± 2.4	6.5 ± 2.2
Insoluble Fiber (g/d)	10.1 ± 4.8	11.0 ± 3.8
Cholesterol (mg/d)	**752.7 ± 123.6**	**201.8 ± 90.2**
Vitamin A	1077 ± 457	1022 ± 551
Vitamin E	9.04 ± 3.67	7.60 ± 4.42
Vitamin D	**7.1 ± 4.1 ^a^**	**3.8 ± 3.4 ^b^**
Selenium	**138.4 ± 54.8 ^a^**	**93.1 ± 41.1 ^b^**
α-carotene (mg/d)	353 ± 448	480 ± 486
β-carotene (mg/d)	2782 ± 2092	3701 ± 3065
Cryptoxanthin	247 ± 596	143 ± 252
Lutein + Zeaxanthin (mg/d)	2226 ± 1835	2600 ± 4222
Lycopene (mg/d)	6000 ± 9099	6026 ± 4656
Physical activity (min)	68.3 ± 47.1	71.3 ± 50.8

^1^ Data are presented as mean ± SD for *n* = 23 subjects. ^2^ Values in the same row with different superscripts (a, b) are significantly different from each other at a *p* values < 0.001. Abbreviations: SFA = saturated fatty acids; MUFA = monounsaturated fatty acids; PUFA = polyunsaturated fatty acids; TFA = trans fatty acids.

**Table 2:** Initial characteristics of subjects.

Parameter	Values
Age (years)	55.2 ± 8.9
Gender Females (%)	65
Waist Circumference (cm)	113.3 ± 12.4
Systolic Blood Pressure (mm Hg)	129.3 ± 7.3
Diastolic Blood Pressure (mm Hg)	87.5 ± 4.8
HDL cholesterol (mg/dL)	47.7 ± 17.4
Triglycerides (mg/dL)	129.5 ± 57.6
Glucose (mg/dL)	102.6 ± 10.5

Values are expressed as mean ± Standard deviation for *n* = 23 subjects.

**Table 3:** Anthropometrics, blood pressure, C reactive protein (CRP), creatinine and liver enzymes, alanine aminotransferase (ALT), aspartate aminotransferase (AST), at baseline (BL) and post (*p*) intervention with egg and choline bitartrate (CB) supplement ^1^.

Parameter	BL-Egg	*p*-Eggs	BL-CB	*p*-CB
Body weight (Kg) ^2^	94.1 ± 19.0	94.0 ± 19.2	94.1 ± 18.6	93.9 ± 18.9
BMI (kg/m^2^)	32.4 ± 4.7	32.3 ± 4.8	32.4 ± 4.6	32.3 ± 4.7
Waist circumference (cm)	112.3 ± 12.0	112.9 ± 11.9	112.7 ± 12.1	112.1 ± 12.3
Systolic blood pressure (mm Hg)	127.6 ± 8.2	127.8 ± 11.7	128.2 ± 7.6	129.5 ± 7.7
Diastolic blood pressure (mm Hg)	85.6 ± 16.3	85.4 ± 7.5	81.8 ± 16.8	85.9 ± 7.0
CRP (mg/dL)	**0.48 ± 0.57 ^a^**	**0.32 ± 0.31 ^b^**	**0.36 ± 0.31 ^ab^**	**0.36 ± 0.34 ^ab^**
ALT (U/L)	27.3 ± 13.2	27.3 ± 13.3	26.8 ± 12.9	24.8 ± 10.2
AST (U/L)	22.9 ± 6.1	23.9 ± 9.1	23.4 ± 7.4	23.9 ± 6.3

^1^ Data are presented as mean ± SD for *n* = 23 subjects. ^2^ Values in the same row with different superscripts (a, b) are significantly different from each other (*p* < 0.01).

**Table 4:** Plasma total, LDL, HDL cholesterol, triglycerides, and glucose in subjects at baseline (BL), post (*p*) intervention with egg and choline bitartrate (CB) supplement ^1^.

Parameter	BL-Egg	*p*-Egg	BL-CB	*p*-CB
Total cholesterol (mg/dL)	177.7 ± 27.3	185.9 ± 25.5	177.9 ± 19.1	185.3 ± 24.8
LDL Cholesterol (mg/dL)	105.4 ± 23.2	111.4 ± 20.5	103.2 ± 16.5	110.8 ± 21.4
HDL Cholesterol (mg/dL)	48.5 ± 16.9	50.2 ± 16.7	48.4 ± 14.3	48.5 ± 14.8
Triglycerides (mg/dL)	122.0 ± 46.9	122.4 ± 46.9	137.3 ± 56.6	129.3 ± 42.2
Plasma Glucose (mg/dL)	100.6 ± 10.8	101.9 ± 10.9	99.8 ± 12.5	101.8 ± 11.3
LDL/HDL ratio	2.43 ± 0.92	2.47 ± 0.97	2.33 ± 0.78	2.50 ± 0.99

^1^ Data are presented as mean ± SD for *n* = 23 subjects.

**Table 5:** Plasma IL-6, TNF-, and MCP-1 in subjects at baseline post (*p*) intervention with egg and choline bitartrate (CB) supplement ^1^.

Parameter	Baseline	*p*-Egg	*p*-CB
Il-6 (pg/mL) ^2^	5.6 ± 0.9 ^a^	4.6 ± 1.7 ^b^	4.4 ± 1.3 ^b^
TNF-α (pg/mL)	5.1 ± 1.1	5.9 ± 1.6	5.9 ± 1.5
MCP-1 (pg/mL)	107.4 ± 46.1	110.9 ± 31.6	116.0 ± 31.8
Insulin (pg/mL)	**152.4 ± 87.2 ^a^**	**97.7 ± 66.5 ^b^**	**111.3 ± 100 ^a,b^**
Insulin Resistance (HOMA)	**5.43 ± 0.38 ^a^**	**3.16 ± 0.26 ^b^**	**4.01 ± 0.40 ^a,b^**

HOMA: Homeostatic Model Assessment. ^1.^ Data are presented as mean ± SD for *n* = 23 subjects. ^2^ Numbers in the same row with different superscripts (a, b) are significantly different from each other (*p* < 0.01). IL-6 = inteleukin-6; TNF-α = tumor necrosis factor- α; MCP-1 = monocyte chemoattractant protein-1.

## References

[1] Grundy S.M., Williams C., Vega G.L. (2018). Upper body fat predicts metabolic syndrome similarly in men and women. Eur. J. Clin. Investig..

[2] Drewnowski A. (2010). The Nutrient Rich Foods Index helps to identify healthy, affordable foods. Am. J. Clin. Nutr..

[3] Rehault S.M., Guyot N., Nys Y. (2019). The Golden Egg: Nutritional Value, Bioactivities, and Emerging Benefits for Human Health. Nutrients.

[4] Wal J.S.V., Gupta A., Khosla P., Dhurandhar N.V. (2008). Egg breakfast enhances weight loss. Int. J. Obes. (Lond.).

[5] Volek J.S., Feinman R.D. (2005). Carbohydrate restriction improves the features of Metabolic Syndrome. Metabolic Syndrome may be defined by the response to carbohydrate restriction. Nutr. Metab..

[6] Zhong V.W., Van Horn L., Cornelis M.C., Wilkins J.T., Ning H., Carnethon M.R., Greenland P., Mentz R.J., Tucker K.L., Zhao L. (2019). Associations of Dietary Cholesterol or Egg Consumption With Incident Cardiovascular Disease and Mortality. JAMA.

[7] Khawaja O., Singh H., Luni F., Kabour A., Ali S.S., Taleb M., Ahmed H., Gaziano J.M., Djoussé L. (2017). Egg Consumption and Incidence of Heart Failure: A Meta-Analysis of Prospective Cohort Studies. Front. Nutr..

[8] Blesso C.N., Andersen C.J., Barona J., Volek J.S., Fernandez M.L. (2013). Whole egg consumption improves lipoprotein profiles and insulin sensitivity to a greater extent than yolk-free egg substitute in individuals with metabolic syndrome. Metab. Clin Exper..

[9] DiMarco D.M., Missimer A., Murillo A.G., Lemos B.S., Malysheva O.V., Caudill M.A., Blesso C.N., Fernandez M.L. (2017). Intake of up to 3 Eggs/Day Increases HDL Cholesterol and Plasma Choline While Plasma Trimethylamine-N-oxide is Unchanged in a Healthy Population. Lipids.

[10] Berger S., Raman G., Vishwanathan R., Jacques P.F., Johnson E.J. (2015). Dietary cholesterol and cardiovascular disease: A systematic review and meta-analysis. Am. J. Clin. Nutr..

[11] Vincent M.J., Allen B., Palacios O.M., Haber L.T., Maki K.C. (2018). Meta-regression analysis of the effects of dietary cholesterol intake on LDL and HDL cholesterol. Am. J. Clin. Nutr..

[12] Kuang H., Yang F., Zhang Y., Wang T., Chen G. (2018). The Impact of Egg Nutrient Composition and Its Consumption on Cholesterol Homeostasis. Cholesterol.

[13] Carson J.A.S., Lichtenstein A.H., Anderson C.A., Appel L.J., Kris-Etherton P.M., Meyer K.A., Petersen K., Polonsky T., Van Horn L. (2020). Dietary Cholesterol and Cardiovascular Risk: A Science Advisory From the American Heart Association. Circulation.

[14] Soliman A.G. (2018). Dietary Cholesterol and the Lack of Evidence in Cardiovascular Disease. Nutrients.

[15] Mistry P., Miller N.E., Laker M., Hazzard W.R., Lewis B. (1981). Individual Variation in the Effects of Dietary Cholesterol on Plasma Lipoproteins and Cellular Cholesterol Homeostasis in Man, Studies of low density lipoprotein receptor activity and 3-hydroxy-3-methylglutaryl coenzyme a reductase activity in blood mononuclear cells. J. Clin. Investig..

[16] McNamara D.J., Kolb R., Parker T.S., Batwin H., Samuel P., Brown C.D., Ahrens E.H. (1987). Heterogeneity of cholesterol homeostasis in man. Response to changes in dietary fat quality and cholesterol quantity. J. Clin. Investig..

[17] Berg J.M., Tymoczko J.L., Stryer L. (2002). The Complex Regulation of Cholesterol Biosynthesis Takes Place at Several Levels. Biochemistry.

[18] Afonso M.S., Machado R.M., Lottenberg A.M., Quintão E.C.R., Moore K.J., Lottenberg A.M. (2018). Molecular Pathways Underlying Cholesterol Homeostasis. Nutrients.

[19] Blesso C.N. (2015). Egg Phospholipids and Cardiovascular Health. Nutrients.

[20] U.S. Department of Health and Human Services and U.S. Department of Agriculture (2015). 2015–2020 Dietary Guidelines for Americans, 8th ed. https://health.gov/our-work/food-and-nutrition/2015-2020-dietary-guidelines/.

[21] Zeisel S.H., Da Costa K.-A. (2009). Choline: An essential nutrient for public health. Nutr. Rev..

[22] Detopoulou P., Panagiotakos D.B., Antonopoulou S., Pitsavos C., Stefanadis C. (2008). Dietary choline and betaine intakes in relation to concentrations of inflammatory markers in healthy adults: The ATTICA study. Am. J. Clin. Nutr..

[23] Wallace T.C., Fulgoni V.L. (2016). Assessment of Total Choline Intakes in the United States. J. Am. Coll. Nutr..

[24] Institute of Medicine Dietary Reference Intakes for Thiamin, Riboflavin, Niacin, Vitamin B6, Folate, Vitamin B12, Pantothenic Acid, Biotin, and Choline (1998). Dietary Reference Intakes for Thiamin, Riboflavin, Niacin, Vitamin B6, Folate, Vitamin B12, Pantothenic Acid, Biotin, and Cholin.

[25] Lemos B.S., Medina-Vera I., Malysheva O.V., Caudill M.A., Fernandez M.L. (2018). Effects of Egg Consumption and Choline Supplementation on Plasma Choline and Trimethylamine-N-Oxide in a Young Population. J. Am. Coll. Nutr..

[26] Yan J., Wang W., Gregory J.F., Malysheva O., Brenna J.T., Stabler S.P., Allen R.H., A Caudill M. (2011). MTHFR C677T genotype influences the isotopic enrichment of one-carbon metabolites in folate-compromised men consuming d9-choline. Am. J. Clin. Nutr..

[27] Matthews D.R., Hosker J.P., Rudenski A.S., Naylor B.A., Treacher D.F., Turner R.C. (1985). Homeostasis model assessment: Insulin resistance and β-cell function from fasting plasma glucose and insulin concentrations in man. Diabetologia.

[28] Blesso C.N., Andersen C., Barona J., Volk B., Volek J.S., Fernandez M.L. (2013). Effects of carbohydrate restriction and dietary cholesterol provided by eggs on clinical risk factors in metabolic syndrome. J. Clin. Lipidol..

[29] Gao X., Wang Y., Sun G. (2017). High dietary choline and betaine intake is associated with low insulin resistance in the Newfoundland population. Nutrition.

[30] Nakamura T., Fujiwara R., Ishiguro N., Oyabu M., Nakanishi T., Shirasaka Y., Maeda T., Tamai I. (2010). Involvement of choline transporter-like proteins, CTL1 and CTL2, in glucocorticoid-induced acceleration of phosphatidylcholine synthesis via increased choline uptake. Biol. Pharm. Bull..

[31] Wang M. (2005). The role of glucocorticoid action in the pathophysiology of the Metabolic Syndrome. Nutr. Metab..

[32] Pinelli N.R., Jaber L.A., Brown M.B., Herman W.H. (2010). Serum 25-Hydroxy Vitamin D and Insulin Resistance, Metabolic Syndrome, and Glucose Intolerance Among Arab Americans. Diabetes Care.

[33] Farrokhian A., Bahmani F., Taghizadeh M., Mirhashemi S.M., Aarabi M.H., Raygan F., Aghadavod E., Asemi Z. (2016). Selenium Supplementation Affects Insulin Resistance and Serum hs-CRP in Patients with Type 2 Diabetes and Coronary Heart Disease. Horm. Metab. Res..

[34] Mäntyselkä P., Kautiainen H., Saltevo J., Würtz P., Soininen P., Kangas A.J., Ala-Korpela M., Vanhala M. (2012). Weight change and lipoprotein particle concentration and particle size: A cohort study with 6.5-year follow-up. Atherosclerosis.

[35] Rosenkilde M., Rygaard L., Nordby P., Nielsen L.B., Stallknecht B.M. (2018). Exercise and weight loss effects on cardiovascular risk factors in overweight men. J. Appl. Physiol..

[36] Guo J., Hobbs D.A., Cockcroft J.R., Elwood P.C., Pickering J.E., Lovegrove J.A., Givens D.I. (2017). Association between egg consumption and cardiovascular disease events, diabetes and all-cause mortality. Eur. J. Nutr..

[37] Pourafshar S., Akhavan N.S., George K.S., Foley E.M., Johnson S.A., Keshavarz B., Navaei N., Davoudi A., Clark E.A., Arjmandi B.H. (2018). Egg consumption may improve factors associated with glycemic control and insulin sensitivity in adults with pre- and type II diabetes. Food Funct..

[38] Djoussé L., Kamineni A., Nelson T.L., Carnethon M.R., Mozaffarian D., Siscovick D., Mukamal K.J. (2010). Egg consumption and risk of type 2 diabetes in older adults. Am. J. Clin. Nutr..

[39] Sharma P. (2011). Inflammation and the Metabolic Syndrome. Indian J. Clin. Biochem..

[40] Devaraj S., Singh U., Jialal I. (2009). Human C-reactive protein and the metabolic syndrome. Curr. Opin. Lipidol..

[41] Ratliff J.C., Mutungi G., Puglisi M.J., Volek J.S., Fernandez M.L. (2008). Eggs modulate the inflammatory response to carbohydrate restricted diets in overweight men. Nutr. Metab..

[42] Mutungi G., Ratliff J., Puglisi M., Torres-Gonzalez M., Vaishnav U., Leite J.O., Quann E., Volek J.S., Fernandez M.L. (2008). Dietary Cholesterol from Eggs Increases Plasma HDL Cholesterol in Overweight Men Consuming a Carbohydrate-Restricted Diet. J. Nutr..

[43] Greene C.M., Zern T.L., Wood R.J., Shrestha S., Aggarwal D., Sharman M.J., Volek J.S., Fernandez M.L. (2005). Maintenance of the LDL Cholesterol:HDL Cholesterol Ratio in an Elderly Population Given a Dietary Cholesterol Challenge. J. Nutr..

[44] Ballesteros M.N., Cabrera R.M., Saucedo M.D.S., Fernandez M.L. (2004). Dietary cholesterol does not increase biomarkers for chronic disease in a pediatric population from northern Mexico. Am. J. Clin. Nutr..

